# Safety and feasibility of cell-based therapy of autologous bone marrow-derived mononuclear cells in plate-stabilized proximal humeral fractures in humans

**DOI:** 10.1186/s12967-016-1066-7

**Published:** 2016-11-15

**Authors:** Caroline Seebach, Dirk Henrich, Simon Meier, Christoph Nau, Halvard Bonig, Ingo Marzi

**Affiliations:** 1 Department of Trauma Surgery, Johann-Wolfgang-Goethe University, Theodor-Stern-Kai 7, Main, 60590 Frankfurt, Germany; 2 Institute for Transfusion Medicine and Immune Hematology, Johann-Wolfgang-Goethe University, and DRK-Blutspendedienst Baden-Württemberg-Hessen, Main, Frankfurt, Germany

**Keywords:** BMC, Bone regeneration, Cell therapy, Proximal humeral fracture, Bone defect

## Abstract

**Background:**

Local implantation of ex vivo concentrated, washed and filtrated human bone marrow-derived mononuclear cells (BMC) seeded onto β-tricalciumphosphate (TCP) significantly enhanced bone healing in a preclinical segmental defect model. Based on these results, we evaluated in a first clinical phase-I trial safety and feasibility of augmentation with preoperatively isolated autologous BMC seeded onto β-TCP in combination with angle stable plate fixation for the therapy of proximal humeral fractures as a potential alternative to autologous bone graft from the iliac crest.

**Methods:**

10 patients were enrolled to assess whether cell therapy with 1.3 × 10^6^ autologous BMC/ml/ml β-TCP, collected on the day preceding the definitive surgery, is safe and feasible when seeded onto β-TCP in patients with a proximal humeral fracture. 5 follow-up visits for clinical and radiological controls up to 12 weeks were performed.

**Results:**

β-tricalciumphosphate fortification with BMC was feasible and safe; specifically, neither morbidity at the harvest site nor at the surgical wound site were observed. Neither local nor systemic inflammation was noted. All fractures healed within the observation time without secondary dislocation. Three adverse events were reported: one case each of abdominal wall shingles, tendon loosening and initial screw perforation, none of which presumed related to the IND.

**Conclusions:**

Cell therapy with autologous BMC for bone regeneration appeared to be safe and feasible with no drug-related adverse reactions being described to date. The impression of efficacy was given, although the study was not powered nor controlled to detect such. A clinical trial phase-II will be forthcoming in order to formally test the clinical benefit of BMC-laden β-TCP for PHF patients.

*Trial registration* The study was registered in the European Clinical Trial Register as EudraCT No. 2012-004037-17. Date of registration 30th of August 2012. Informed consent was signed from all patients enrolled.

## Background

Large bone defects after severe trauma, debridement of pseudarthrosis and osteomyelitis, or osteoporotic fractures remain a major challenge in trauma and orthopedic surgery due to impaired vascularization and delayed bone healing. Current treatment protocols recommend the use of autologous and allogenous bone to repair these defects but are fraught with high donor site morbidity, reduced biological capacity, limited availability of autologous bone, immunogenicity or loosening of alloplastic implants and extended surgical times [1]. Tissue engineering is an attractive alternative approach to current conventional treatment protocols and could help to minimize or eliminate the limitations and/or costly complications of the latter.

Bone tissue engineering combines cells with regenerative potential, synthetic or natural osteoconductive scaffolds (e.g. β-tricalciumphosphate, β-TCP) and biological factors [2, 3]. Previously, we demonstrated that cell based therapy using extensively cultivated stem cells (mesenchymal stem cells, MSC, endothelial progenitor cells, EPC) implanted on a β-TCP scaffold into a femoral large-sized segmental bone defect in rats leads to improved vascularization and new bone formation in the defect site [4–6]. Albeit efficient, the use of long-term cultured progenitor cells is fraught with disadvantages like delay of definitive surgery, questions of biological safety, high costs and is not yet established clinically. Tissue engineering approaches using fresh autologous bone marrow mononuclear cells (BMC) might circumvent many of those limitations. Human BMC can be harvested and reintroduced to the patient within hours which is more compatible with the clinical requirement for rapid definitive fracture fixation. BMCs are already used in cardiology as well as in vascular surgery and are ideally suited for regenerative medicine due to their apparent regenerative potential and safety profile [7, 8]. Using a femur defect model in rats we previously demonstrated that BMCs combined with a β-TCP scaffold enhanced the bone healing response. The histological quality and the mechanical strength of femur defects treated with BMC were qualitatively comparable to those defects receiving cultured MSC and EPC [9].

Based on those promising results, we aimed to establish a cell-based bone regeneration procedure applicable in the whole field of bone defects after trauma, tumors, joint arthroplasty and in osteoporotic defects. We hypothesized that transplantation of BMC + β-TCP into a large bone defect should be safe, feasible and should promote bone formation and bony bridging of the defect resulting in improved clinical outcomes.

Therefore, we investigated safety and feasibility of augmentation with preoperatively isolated autologous BMC cells seeded onto β-TCP in combination with angle stable fixation (Philos plate®) for the therapy of proximal humeral fractures (PHF) in a clinical phase-I trial. This frequent fracture type is characterized by a high rate of secondary dislocation (varus collapse of the humeral head of more than 20° or screw perforation) and complication rate up to 30% [10]. Thus, this fracture is suitable to study in a standardized clinical situation bone healing by autologous bone marrow cell transplantation seeded onto an established scaffold.

## Patients and methods

### Objectives

This was a clinical phase-I trial to test safety and feasibility of augmentation with preoperatively isolated autologous BMC cells seeded onto β-TCP in combination with angle stable fixation (Philos plate®) for the therapy of proximal humerus fractures.

#### Ethics and regulatory affairs

A manufacturing license for tissue procurement acc. to §20b German Medicines Law and for manufacturing of the advanced therapy medicinal product (ATMP) “BMC2012” (see below) acc. to §13 German Medicines Law, the autologous cell-based study drug, was obtained from the local regulatory agency (Regierungspräsidium Darmstadt). Protocols for a German Medicines Law GCP trial were prepared and permissions from the local Ethics board [No. 350/12] and the federal autority (PEI) [No. 1769] were obtained for treatment of 10 consecutive, eligible, consenting patients. The study was registered in the European Clinical Trial Register as EudraCT No. 2012-004037-17. Informed consent was signed by all participants. No study-specific X-rays were performed.

#### Inclusion/exclusion criteria

Criteria for inclusion were 2-, 3- or 4-fragment fracture (Neer classification), dislocation of ≥10 mm between fragments and/or angle of ≥45° between fragments and/or dislocation of tuberculum major of ≥5 mm, age >18 years, informed consent for surgery and study participation.

Exclusion criteria were pregnancy, luxation fracture, nerve damage, progressive tumor disease and mental-health problems or other causes for inability to provide informed consent.

### Experimental group/control group

This was a single-arm uncontrolled study. All patients received cell-based therapy with BMC: open reduction internal fixation (ORIF) of the fracture, augmentation with composite of an acellular bone graft substitute (ß-TCP) and 1.3 × 10^6^ BMC/ml TCP. Epidemiological data of patients are shown in Table 1.

**Table 1:** Patient epidemiology

Patient	Age	Gender	Smoker	Osteoporosis
1	69	Female	No	No
2	71	Female	No	No
3	69	Female	Yes	Yes
4	67	Female	No	No
5	64	Female	No	No
6	72	Male	No	No
7	76	Female	No	No
8	66	Female	No	No
9	71	Female	No	No
10	66	Female	No	No

### Investigational new drug (IND): BMC2012

#### Collection, manufacture, testing and release of the cell product BMC

On the day prior to surgery, 50 ml bone marrow, anti-coagulated with heparin, were aseptically aspirated from the posterior iliac crest under local anesthesia in regulator-approved intervention rooms. Additionally, 27 ml peripheral blood were aseptically collected into endotoxin-free “no additive” vials. Bone marrow aspirate and blood were transported immediately under standardized conditions (20 ± 2 °C) to the Department of Transfusion Medicine and DRK Blutspendedienst, Frankfurt. The blood samples in the serum vacuettes remained with the bone marrow aspirate at all times and were used to prepare autologous serum under GMP conditions. For this purpose clotted blood was centrifuged at 2800*g* for 15 min. The serum was aseptically aspirated (clean room class A in B) and used for final drug preparation.

BMC preparation was performed under full GMP in the certified facility of the Department of Transfusion Medicine as described in [11]. Briefly, BM aspirate was diluted in saline and subjected to Ficoll (Lonza, Verviers, Belgium) density purification. The interphase cells were carefully collected, pooled, washed and re-suspended in ex vivo (Lonza) containing 20% v/v autologous serum. All open and semi-open steps were performed in a class A in B, all other steps in a class B environment. BMC were counted, diluted to a final concentration of 1.3 × 10E6/ml suspension media and aseptically transferred to a cryostorage bag (Miltenyi Biotech, Bergisch-Gladbach, Germany). The final product consisted of 12 ml BMC suspension; it was stored at room temperature until use. The IND specification was as following: WBC concentration 1.3 × 10e6 ± 10%/ml, CD34 + cell concentration measured and declared, CD45+ cell viability >95%, bioburden negative-to-date, donor negative by serology for Hepatitis A, B, C, Syphilis and HiV. Tests were performed using the following assays: Total leukocytes (WBC) were enumerated using the Sysmex XT1800 hemacytometer (Norderstedt, Germany). Content of putative progenitor cells (CD271+/CD73+/CD45− (putative MSC); CD45+/CD34+/CD133+/VEGFR2+ (putative EPC); CD45+ /CD34+ /CD133+ (putative HSPC) was determined by single-platform flow cytometry using Trucount counting beads (Becton–Dickinson, Heidelberg, Germany) and a dual-laser FACS Calibur (Becton–Dickinson). A sample for bioburden assessment was taken by overfilling the final product bag by one mL and withdrawing one mL half of which was subsequently inoculated each onto aerobic and anaerobic BacT/Alert bottles (BioMerieux, Nürtingen, Germany [12].

**Fig. 1:**
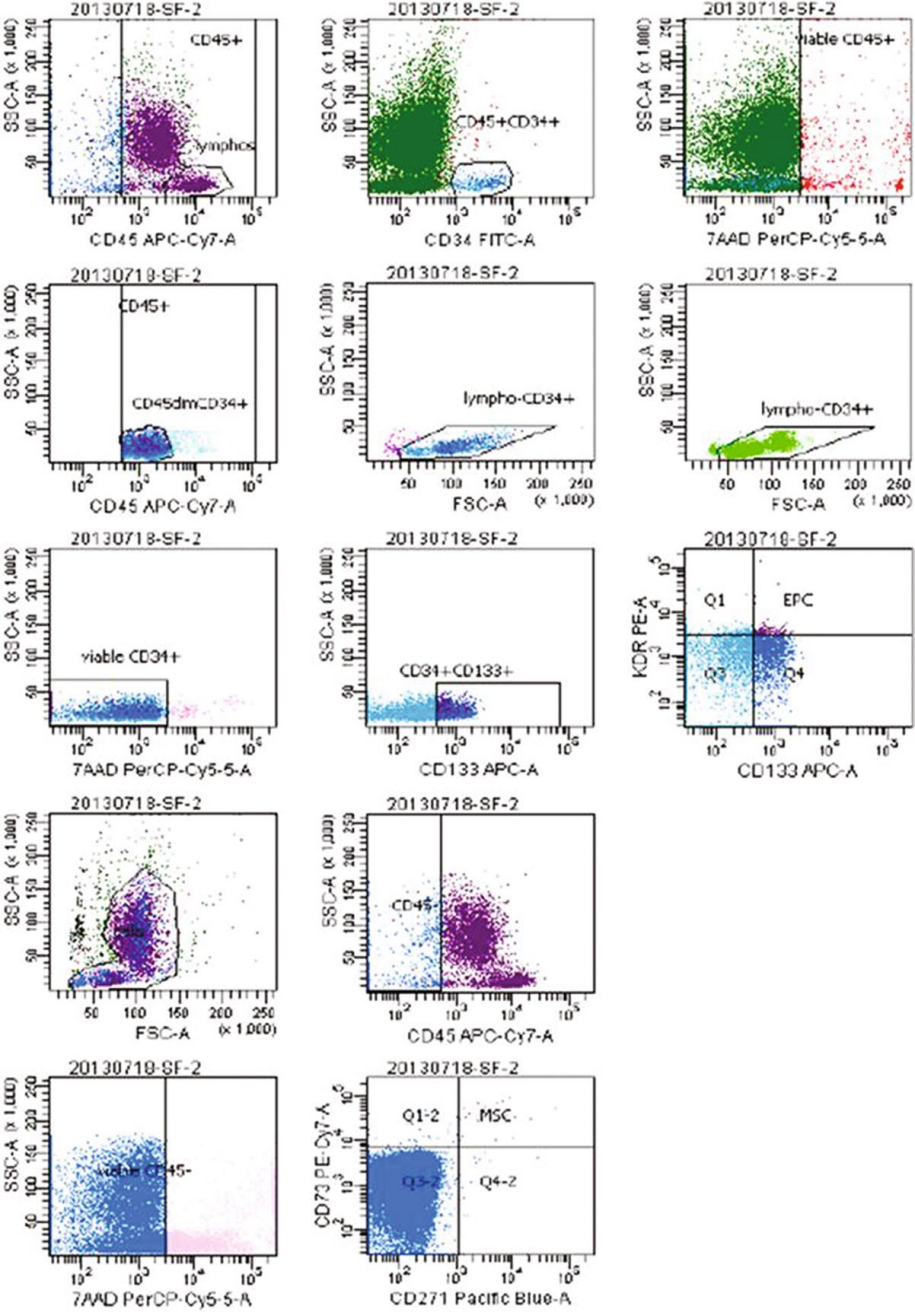
HSCs were recognized as 7AAD-negative, CD34+ /CD45dim/SSC-lo/FSC-lomid events. (*PANEL 1*, not shown) using an IVD-grade commercial HSC enumeration platform, SCE (BD, Heidelberg, Germany). The ISHAGE panel was subsequently extended to contain antibodies against CD133 and KDR for recognition of putative EPCs among the ISHAGE-HSCs, and against CD73 and CD271 for recognition of putative MSCs among the CD45-negative cells (*PANEL 2*, shown here)

HSCs were recognized following the ISHAGE convention, as also laid out in the Eur. Pharm., as 7AAD-negative, CD34+ /CD45dim/SSC-lo/FSC-lomid events (PANEL 1, not shown), using an IVD-grade commercial HSC enumeration platform, SCE (BD, Heidelberg, Germany) [13]. The ISHAGE panel was subsequently extended to contain antibodies against CD133 and KDR for recognition of putative EPCs among the ISHAGE-HSCs, and against CD73 and CD271 for recognition of putative MSCs among the CD45-negative cells (PANEL 2, see Fig. 1). HSC enumeration was validated against the SCE kit (HSC frequency among CD45+ to within 10% of each other throughout the relevant frequency range), in order to allow calculation of EPC and MSC concentrations based on HSC concentration in PANEL 1 and relative frequency of EPC and MSC vs. HSC in PANEL 2.

Quality control further included assessment of vitality (7AAD). All release-critical assays used were validated according to guidelines laid out in the European Pharmacopoiea.

### Ancillary tests on the IND

#### Assessment of maximum storage time

With permission of the ethics committee, residual cell suspension not needed for formulation of the study drug was used for further analyses. Thus five (four for CFU-F) residual patient samples were analysed over time to assess the shelf-life of the BMCs under the clinically relevant storage conditions (X-Vivo10 w/20% autologous serum, room temperature, not gas-permeable storage bag).Flow cytometry was applied to determine alterations of the frequencies of some putative progenitor cell populations (putative MSC, HSPC; CD45+/CD34+/CD133±) over time (24, 48 and 72 h after BMC isolation) as described in [14].

Colony-forming units-fibroblast (CFU-F) were enumerated determined using the CFU-F-assay as described in [14].

#### Estimation of the seeding efficacy

The single step procedure for BMC seeding on β-TCP scaffold material mimicking the clinical intraoperative procedure was applied. Residual BMC from study participants [1.33 × 10^6^ BMC/ml medium, n = 5] were dripped onto densely packed β-TCP scaffold material (granule size 1.4–2.8, 1.4–2.8 mm, *Chronos*, Synthes) in a cell strainer with 100 µm pore size (BD-Biosciences, Heidelberg, Germany). Non-adhering cells were collected at the bottom of the test tube, counted and the seeding efficacy was calculated.

### Application of the IND

Within 24 h of marrow aspiration, BMC were applied during surgery and plate osteosynthesis of the fractures. Briefly, the large bone defect was bridged following the clinical standard. Subsequently, the defect was filled with a clinically established β-TCP scaffold (size: 1.4–2.8 mm, *Chronos*, Synthes, Dubendorf, Switzerland), and an equal volume of BMC suspension was loaded carefully in situ on the implanted β-TCP using a syringe. The phenotype of BMC adhering to the scaffold was analyzed previously [14].

### Follow-up per patient

Each study participant underwent five visits for study purposes and monitoring of safety/tolerability and bone healing over a period of 12 ± 2 weeks as depicted in Table 2.

**Table 2:** Patient flow chart of the clinical trial

	Visit 1	Visit 2	Visit 3	Visit 4	Visit 5
Timepoint	24 h before surgery, study inclusion, BMC aspiration from iliac crest	Surgery, implantation of BMC	1–7 days after surgery	Within 6 ± 1 weeks after surgery	Within 12 ± 2 weeks after surgery
BM aspiration	×				
Implantation of BMC		×			
Inflammation (local)	×	×	×	×	×
Inflammation (WBC, CRP, IL-6, PCT) fever	×	×	×	×	×
Evaluation of AEs		×	×	×	×
Evaluation of medication	×	×	×	×	×
Radiology	×	×	×	×	×
Functional assessment (dash-score)					×

### Endpoints

Primary endpoints were safety and feasibility. To objectively assess these, morbidity of cell harvesting procedure, the occurrence of local infection at the fracture site after cell transplantation (inflammation, wound healing disruption) as well as systemic inflammation (WBC, CRP, IL-6, PCT) and fever (>38.5 °C) for more than 2 days were documented during the follow up period.

With regard to feasibility of the procedure of bone marrow harvesting, logistics for BMC preparation and transport as well as explanatory power of the clinical controls relating to the possible clinical benefit were evaluated.

As secondary endpoints bone healing (X-ray), clinical functional outcome (DASH score), evaluation of medication and AEs were assessed.

### Evaluation of bone healing

**Fig. 2:**
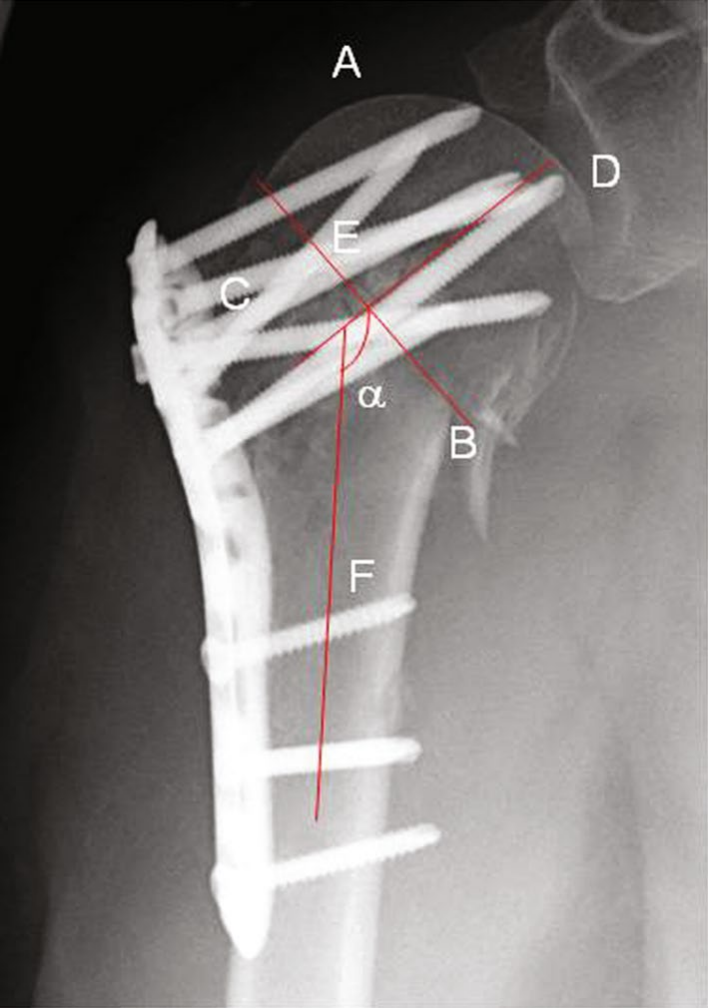
Radiological assessment of humeral position after surgical repositioning and during convalescence: A line from the upper to the lower limit of joint surface (*A*–*B line*), and a* perpendicular line* to* A*–*B* were drawn through the center of the humeral head (*C*–*D line*). The angle alpha between this* line* and the* bisecting line* of the humeral shaft (*E*–*F line*) was called head-shaft-angle. The distance between the screw top and the joint surface (d) was also measured

The “true anterior-posterior (true a.p.)” and “outlet-view” X-rays of the shoulder were performed at Visit 1–5 according to clinical standard in order to evaluate fracture site and implant position as well as to detect screw-cutting-out, osteonecrosis, pseudarthrosis and loosening of implants and definitive bone healing. Due to fracture-immanent possible secondary varus dislocation we measured head-shaft-angle in true a.p. X-ray. Therefore, a line from the upper to the lower limit of joint surface was drawn (A–B line), then a perpendicular line to A–B line through the center of the humeral head (C–D line). The angle alpha between this line and the bisecting line of the humeral shaft (E–F line) was measured as head-shaft-angle (Fig. 2).

Secondary dislocation was defined as a secondary loss of reposition result of ≥20° of head-shaft-angle in the true a.p.

In order to detect a secondary screw-cutting-out we also measured the distance between the top of the screws to the joint surface (d).

### DASH score

Twelve weeks postoperatively movement and function of the shoulder were evaluated by disabilities of the arm, shoulder and hand score (DASH) [15].

Therefore patients had to respond to thirty questions of a questionnaire about everyday function of the shoulder within the last week. Then the DASH-Score was measured. The DASH score gives evidence to function, symptoms and special activity (athlete, musician). A DASH-score of 0 is a result with an optimal function without limitation. A DASH score of 100 means a maximal limitation.

### Evaluation of medication and AEs

Medication and AEs were documented at each study visit (V1–5) in descriptive manner.

#### Statistics

In this phase-I clinical trial safety and feasibility of cell based therapy by implanted bone marrow-derived mononuclear cells (BMC) for bone augmentation of plate-stabilized proximal humeral fractures were tested. Ten patients were planned and included in the study, all of whom received BMC 2012. Data were presented descriptively. Thus no statistical comparison was performed regarding neither data of safety and feasibility nor data of secondary endpoints (bone healing, DASH score).

Data for cell experiments are evaluated statistically by non parametric Wilcoxon matched pair analysis, a *p* value below 0.05 indicates statistical significance. Results were presented either as box plots of the median (box: median, 25% quartile and 75% quartile; whiskers: minimum, maximum) or in text, respectively tables as median (25% quartile/75% quartile).

## Results

We generated formal study protocols, including IMPD, and applied for §40 AMG permission from the PEI for this phase I trial (EudraCT-Nr.:2012-004037-17, Date of registration: 30th of August 2012; Date enrolled first participant: 11th of September 2013).

After regulatory approval 10 patients were recruited and completed follow-up between September 2013 and 2014. Epidemiological information of patients is shown in Table 1.

### Primary endpoints

For the endpoint safety/feasibility, morbidity from bone marrow aspiration, local infection at BM aspiration or surgery site, systemic inflammation and fever were investigated.

**Fig. 3:**
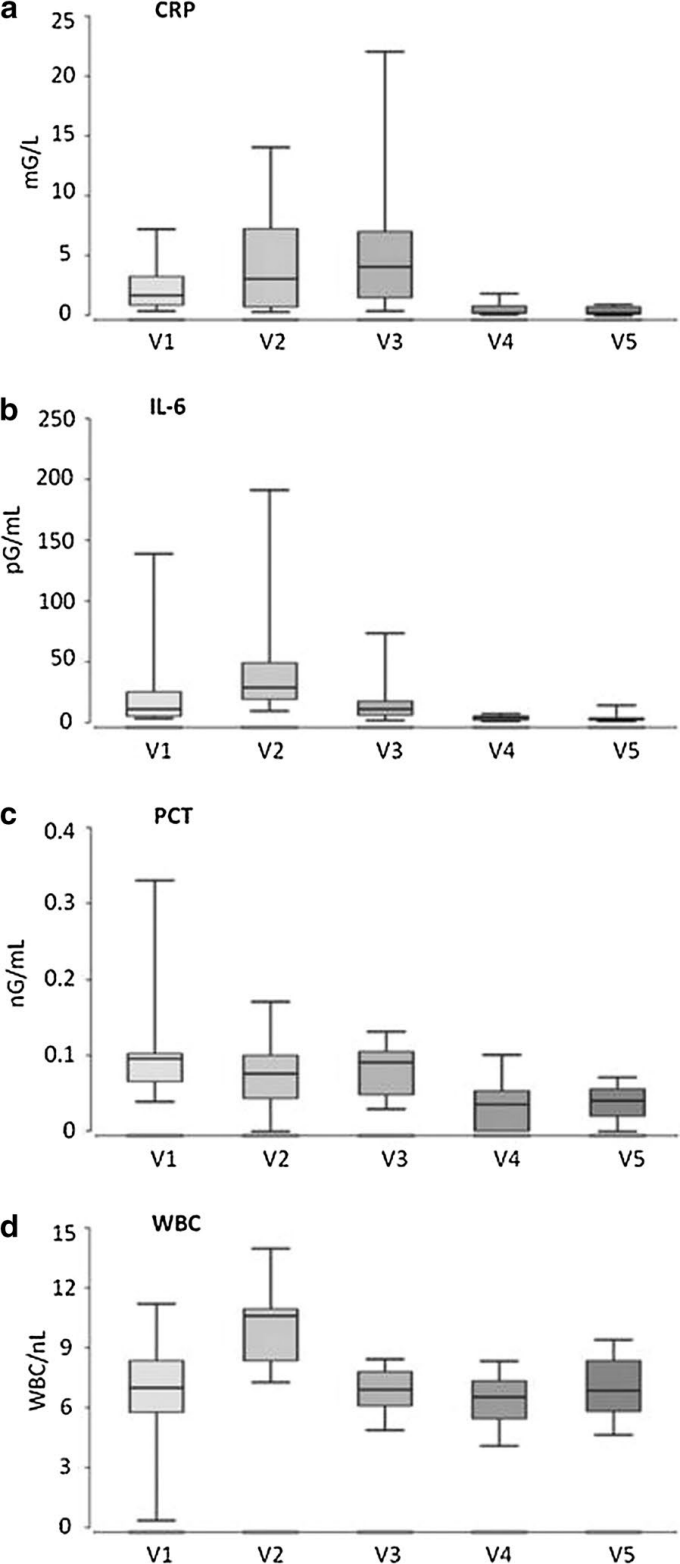
**a**–**d** Blood parameters of systemic inflammation, C-reactive protein (CRP), interleucin-6 (IL-6), procalcitonin (PCT) and white blood cells (WBC) were measured at every five visits (V1–5). The expected initial increase of CRP- and IL-6-values after surgery was observed; values subsequently decreased to within the normal range beyond week 1. *Box plots* of the median (*box*: median, 25% quartile and 75% quartile; *whiskers*: minimum, maximum)

No side effects of bone marrow aspiration were seen. Even though suffering from a humeral fracture, bone marrow aspiration under local anesthesia was well tolerated by all patients. Furthermore, neither local inflammation nor disruption of wound healing process as signs of local infection were observed at any time. Regarding the blood parameters that might indicate systemic inflammation, CRP, IL-6, PCT and WBC were measured at all five visits. The expected initial increase of CRP- and IL-6-values after surgery was observed and subsequently decreased to normal homeostatic values after 1 week. Further, no systemic inflammation was diagnosed by measurement of procalcitonin (Fig. 3a–d). During the whole time no fever was reported.

With respect to feasibility, bone marrow harvesting, manufacturing within the narrow time slot and transport logistics were checked.

Bone marrow aspiration was feasible and well tolerated by all patients. Also the overall logistical implementation of bone marrow aspiration, transportation, production and final application of BMC during surgery and plate osteosynthesis of the fractures was well realizable within the short approved shelf life of the study drug.

### Secondary endpoints

The secondary endpoints were bone healing assessed by X-ray, clinical functional outcome by means of the DASH-score, evaluation of medication and AEs.

**Fig. 4:**
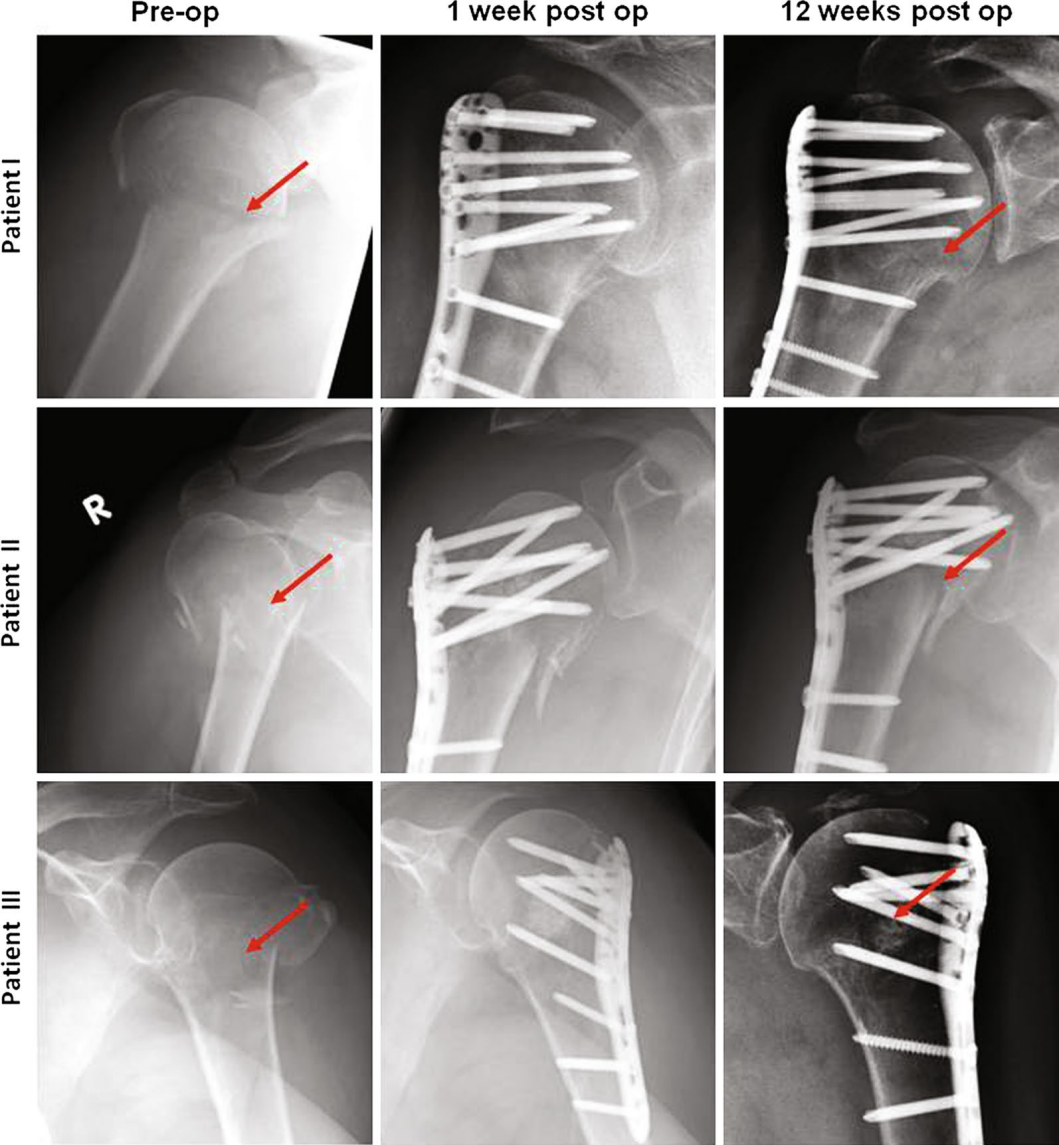
Radiological controls (X-ray) of three representative patients preoperatively, 1–12 weeks after surgery. *Red arrows* show sites of fracture during observation time

In all 10 cases bone fracture healed within the observation time of 12 weeks (Fig. 4). During this period no secondary dislocations of the proximal subcapital humerus fracture were detected by radiological assessment of the head-shaft-angle (140.4° ± 2.6). In addition, no secondary screw perforation due to collapse of the humeral head or the osteosynthesis was noted on the X-ray images.

During study visits only three adverse events were reported. One patient each showed single-segment abdominal wall shingles, partial loosening of the tendon of the supraspinatus muscle and a surgical complication of an initial screw perforation. Solely in one case hospitalization was necessary in order to surgically reattach a supraspinatus tendon, which was consequently classified as a serious adverse event, albeit presumably not related to study drug, which was applied in the subcapital metaphyseal area and not the tendon attachment site.

**Table 3:** Antibodies used for characterization of putative stem/progenitor cell populations within the BMC preparation

Antigen	Conjugation	Company	Catalog#	Clone
CD34	FITC	BD-Biosciences	555820	581
CD45	PerCP	BD-Biosciences	340665	2D1
CD133	PE	Miltenyi biotech	130-080-801	AC133
CD271	APC	Miltenyi biotech	130-091-884	ME20.4-1.H4

The clinical functional outcome by evaluation of the DASH-score after 12 weeks resulted in a value of 52 ± 7.9 (mean ± SEM). It should be noted that the DASH-score is not only influenced by bone healing, but also by cartilage lesions or tendon issues, which are often observed in this age group [16].

#### Loss of CFU-F frequency and reduction of BMC viability after 48 h

**Fig. 5:**
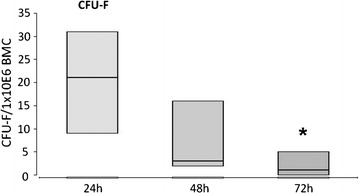
Assessment of maximum storage time. CFU-F assay were used to determine Colony-forming units-fibroblast (CFU-F). *Box plots* of the median (*box*: median, 25% quartile and 75% quartile). *Asterisk* means a significant decline of CFU-F (marrow stromal cells) at 72 h after bone marrow aspiration

The viability of mononucleated cells, the frequency of putative hematopoietic stem cells (CD45+ CD34+ CD133+; CD45+ CD34+ CD133−) as well as of putative marrow stromal cells (CD45^dim^CD271+) were assessed by means of flow cytometry or CFU-F assay (marrow stromal cells) at 24 h (transplantation), 48, and 72 h after bone marrow aspiration (Table 3; Fig. 5). Viability declined significantly from 24 to 48 and 72 h (p < 0.05, Table 4). Correspondingly, the number of viable BMC being calculated in relation to the 24 h-values dropped significantly from 24 to 72 h (p < 0.05, Table 4). In contrast, the frequencies of putative hematopoietic stem cells as well as of putative marrow stromal cells remained constant during the whole observation period, indicating equal sensitivity to storage lesion of all cellular subsets. No significant differences were seen between all time points. This result indicates that the absolute number of stem cells dropped consistently with the absolute BMC numbers (otherwise an enrichment or decline of stem cell frequency would have been seen).

**Table 4:** Storage lesion of the IND (ancillary studies on the IND, to determine maximum shelf life)

Parameter	24 h	48 h	72 h	p values (24 vs 28 h)	p values (24 vs 72 h)
Cell number [absolute values]	1.7E7 (1.9E7/7.7E6)	1.6E7 (1.7E7/1.1E7)	1.1E7 (1.6E7/1.0E7)	p = 0.74, not significant	p = 0.31, not significant
Cell number [% 24 h]	100 (100/100)	95 (100/77.9)	74.1 (87/64.7)	p = 0.17, not significant	p = 0.005, significant
Vital cells [% BMC]	98.8 (98.8/98.4)	96.9 (96.9/96.9)	92.21 (94.0/84.1)	p = 0.11, not significant	p = 0.043, significant
Vital cells [% 24 h]	100 (100/100)	98.0 (98.9/97.1)	94.4 (97.3/85.2)	p = 0.06, trend	p = 0.003, significant
CD34+ CD45+ [% BMC]	1.33 (1.95/1.13)	1.47 (1.85/1.24)	1.32 (1.36/1.15)	p = 1.0, not significant	p = 0.81, not significant
CD34+ CD45+ CD133+ [% BMC]	0.66 (0.75/0.04)	0.6 (0.95/0.43)	0.55 (0.65/0.5)	p = 1.0, not significant	p = 1.0, not significant
CD45− CD271+ [% BMC]	0.01 (0.03/0.01)	0.03 (0.03/0.03)	0.02 (0.03/0.01)	1.0, not significant	1.0, not significant
CFU-F [1.0E6 BMC]	21.0 (1.95/1.13)	3.0 (9.5/2.5)	1.0 (3.0/0.5)	p = 0.1, trend	p = 0.04, significant
CFU-F [% of 24 h]	100.0 (100/100)	22.0 (9.7/76.2)	3.2 (0/23.8)	p = 0.04, significant	p = 0.03, significant

In contrast, absolute values of CFU-F frequency declined in trend from 24 to 48 h (p = 0.1) and significantly from 24 to 72 h (p < 0.05). If CFU-F concentration is calculated as percent change to 24 h-values a significant decline was seen between 24 and 48 h (p < 0.05) respectively 24 and 72 h (p < 0.05, Table 4).

#### Estimation of seeding efficacy

The mean seeding efficacy was 59% ± 8.5 (mean ± SEM) applying a single step seeding procedure mimicking the situation in situ as described in the materials and methods section.

## Discussion

We analyzed the safety and feasibility of autologous BMC in a clinical phase-I trial as a therapeutic option for the treatment of bone defects at high risk of secondary dislocation in ten patients with a proximal humerus fracture. Both aspects, safety and feasibility, could be unequivocally demonstrated. A near-identical BMC production process is already approved by the German federal regulatory authority (Paul-Ehrlich-Institute, Germany) and established at the German Red Cross-Blood Service (DRK-BSD, Frankfurt) for the application of BMC in acute myocardial infarction and limb ischemia [7, 8]. Albeit applied by a different route (i.e.) and for an entirely different clinical indication, in at least six cardiovascular clinical trials the drug was found to be safe and probably efficacious, as well as the logistics of re-applying the cells within 48 h of marrow collections were shown to be feasible on a routine basis [11, 17–22]. The novelty of our approach dictates that similar reports about safety and feasibility of purified BMC for the treatment of bone defects are not yet available.

However, other treatment options ultimately based on the transplantation of vital bone derived cells respectively bone material within the operative procedure by direct separation were previously evaluated by other groups [23, 24]. It needs to be noted, that the large majority of patients still receive complete cancellous bone graft from iliac crest or femur [25], which has the disadvantage of donor site morbidity and limited material. Other approaches such as the use of nonviable scaffolds [26] cannot demonstrate a sufficient biological activity and guided bone healing. Thus, the advantages of using minimally manipulated cell drugs as opposed to ex vivo cultivated stem cells are apparent; these include the risk of transmitting infectious agents with the cells, high laboratory costs and the risk, although probably small, of malignant transformation of long term cultured cells [27–29].

Alternative approaches to bone marrow processing for enrichment of vital progenitor cells have also been taken. Thus concentrated autologous bone marrow aspirate was implanted together with a scaffold consisting of hydroxyapatite into bone defects and reportedly lead to a significant bone healing in almost all cases [30]. Of note, although clearly fulfilling the criteria of an advanced therapy medicinal product (ATMP) and hence requiring a manufacturing authorization and some kind of marketing authorization, these cell products were not regulator-approved at that time.

In humans the expected rate of secondary dislocations after angle-stable fixation of proximal humerus fractures with bone defects has been reported in the literature to range between 20 and 30% [10, 31]. These data are in agreement with the observed outcomes in our routine clinical practice [31].

In our presented clinical phase I study the absence of secondary dislocations suggests a beneficial effect of the BMC on bone healing and provided the rationale for a recently initiated placebo controlled, 1:1 randomized phase II clinical trial (Eudra-CT-No.: 2015-001820-51, ClinicalTrials.gov Identifier: NCT02803177). The advantage of our approach, if proven clinically effective, are the almost immediate availability of the BMC, permitting definitive surgical management of the fracture, the simplicity, and hence affordability, of drug generation, its carefully controlled properties.

### Seeding of BMC on scaffolds in situ

Our clinical protocol restricts the BMC application to a single step in situ seeding procedure on to the freshly implanted β-TCP scaffold due to complex regulatory considerations. In a recent study we evaluated whether a surface coating of the β-TCP scaffold used also in the present study, would enhance BMC adhesion. A three step seeding procedure of BMC was applied and the seeding efficacy was 95% regardless of the surface coating. Further analysis revealed that the seeding procedure is not associated with a cell type specific enrichment or depletion of putative hematopoietic stem cells and putative MSC [14]. Based on those results uncoated β-TCP was used as scaffold in this clinical phase-I trial. Accompanying analyses using residual BMC of the study participants were performed to estimate the seeding efficacy using a one-step seeding procedure in vitro. A mean seeding efficacy of 59% was observed which is significantly lower compared to the three step seeding procedure applied in [9, 14] and lead probably to a reduced number of BMC on the scaffold placed in the defect site. It is a matter of speculation that the reduction of the BMC concentration in the defect adversely affects the bone healing process, keeping in mind that evidence for a correlation between MSC concentration in the defect and the result of the bone healing process was observed [32]. On the other hand, one might hypothesize that the concentration of transplanted BMC within the defect was sufficient since all fractures healed within 12 weeks.

Our in vitro analyses indicate a loss of CFU-F activity during storage. Furthermore, viability is significantly attenuated after 72 h storage at room temperature whereas the frequency of progenitor cell populations among viable cells did not differ significantly. For that reason, the shelf life of *BMC2012* is limited to 48 h after isolation. The decline of CFU-F with increasing storage time was previously reported by Gastens et al. [33]. Hence, it is reasonable to assume that the time between bone marrow harvest and subsequent BMC transplantation might be a critical factor for future BMC-supported therapies of large bone defects and needs to be further addressed.

It seems that BMC constitute powerful candidate cell types for bone regeneration. This cell-based approach seems feasible in clinical settings as well: Cells are easy to harvest, to isolate, to characterize and to provide in a sufficient cell number within some hours. Therefore, if this cell approach could be applied in a human clinical setting, it would clearly improve the present clinical approaches, which are still affected by complications. Complications lead to additional surgeries, high morbidity and loss of working time. Hence, this present biological approach seems feasible, safe and viable.

## Conclusions

Cell therapy with autologous BMC is safe and feasible, as well as probably efficacious when seeded onto β-TCP in situ in patients with proximal humeral fractures, thus a forthcoming clinical trial phase-II is needed.

## Abbreviations

7-AAD: 7-Aminoactinmycin
AE: adverse event
AMG: Arzneimittelgesetz (pharmacia law)
ATMP: advanced therapy medicinal product
BM: bone marrow
BMC: bone marrow-derived mononuclear cells
BMC2012: name of IND
CD45/34/271/133: cluster domain 45 etc.
CFU-F: colony forming units of fibroplasts
CRP: c-reactive protein
DASH score: disabilities of the arm, shoulder and hand
DRK-BSD: deutsches rotes kreuz blutspendedienst (german red cross-blood service)
EPC: endothelial progenitor cells
GMP: German Medicines Law
HSPC: hemtapoietic stem-/progenitor cells
IL-6: interleukin-6
IMPD: investigational medicinal product dossier
IND: investigational new drug
MSC: mesenchymal stroma cells
ORIF: open reduction internal fixation
PCT: procalcitonin
PEI: Paul-Ehrlich-Institute (German federal regulatory authority)
PHF: proximal humerus fracture
TCP: tricalciumphosphate
WBC: white blood count
